# Supplementary material for: MRN complex-dependent recruitment of ubiquitylated BLM helicase to DSBs negatively regulates DNA repair pathways
Source: Nat Commun. 2018 Mar 9;9:1016. doi: 10.1038/s41467-018-03393-8 (PMC5844875; doi:10.1038/s41467-018-03393-8)
Supplement: Supplementary file 1 — Supplementary Information [file 41467_2018_3393_MOESM1_ESM.pdf]

SUPPLEMENTARY INFORMATION

MRN complex-dependent recruitment of ubiquitylated BLM helicase to DSBs negatively regulates DNA repair pathways

Tripathi et al.

Supplementary Figure 1
(Tripathi et al.)

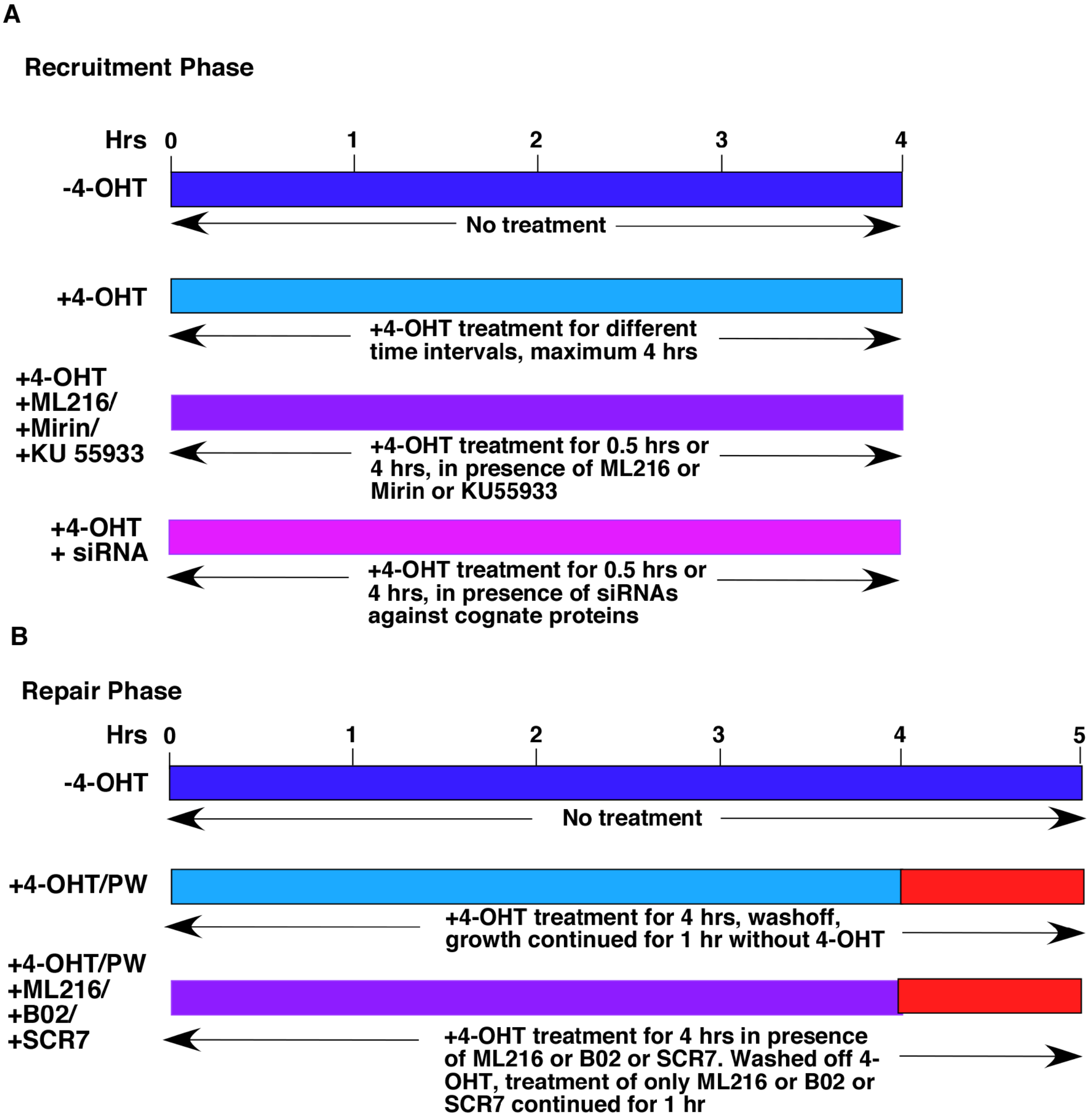

Schematic diagram regarding the conditions of cell growth for recruitment and repair studies. U2OS AsiSI-ER cells were grown in the conditions mentioned for (A) Recruitment phase (B) Repair phase. U2OS-AsiSi-ER cells were either treated with only the respective vehicles (-4-OHT) or treated with 4-OHT. Depending on the experiment, cells in -4-OHT condition were treated with equal volume of the solvent in which ML216, Mirin, KU 55933, B02 or SCR7 were dissolved. 4-OHT treatment was for the indicated time points with a maximum of 4 hrs. Cells were treated with Mirin and KU55933 for 1 hr prior to and during 4-OHT treatment (either 0.5 hr or 4 hrs). 4-OHT treatment was for 0.5 hr or 4 hrs for the siRNA experiments. ML216 treatment was carried out for 24 hrs prior to and during 4-OHT treatment. B02 and SCR7 treatments were carried out during 4-OHT treatment for the last 3 hrs. For the repair phase 4-OHT treatment was for 4 hrs after which it was washed off. The extracts were made 1 hr after 4-OHT was washed off during which the respective drugs (ML216, B02, SCR7) continued to be present in the medium.

Supplementary Figure 2
(Tripathi et al.)

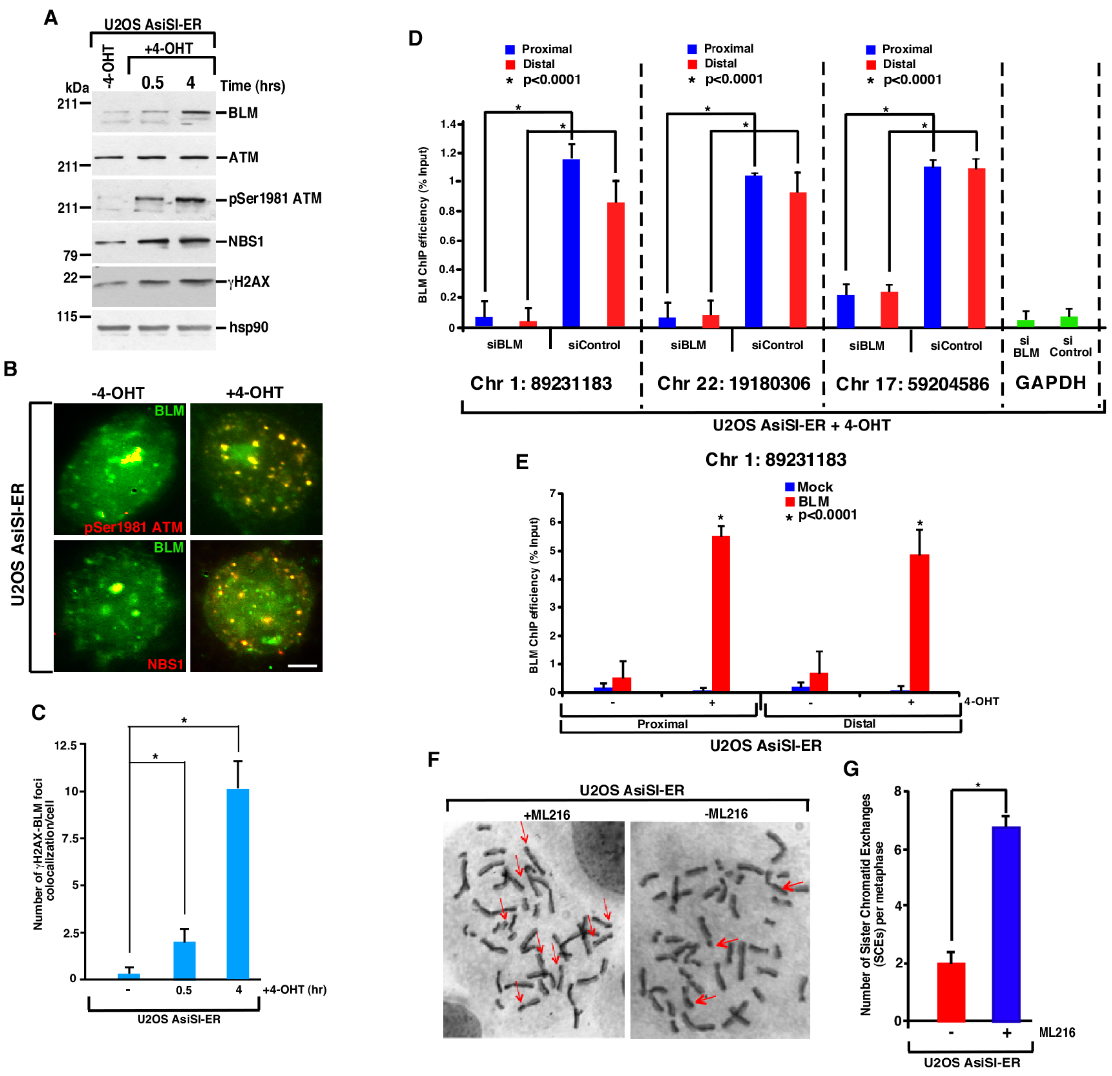

A. BLM and DDR proteins are stabilized after induction of DSBs. U2OS AsiSI-ER cells were either grown in asynchronous condition (-4-OHT) or treated with 4-OHT (+4-OHT) for the indicated time periods. Western blots were carried out with the corresponding lysates using antibodies against BLM, ATM, pSer1981ATM, NBS1, γH2AX and hsp90.

B. BLM foci localizes with DDR proteins after DSB induction. U2OS AsiSI-ER cells were either grown in asynchronous condition (-4-OHT), or treated with 4-OHT (+4-OHT) for 4 hrs. Cells were fixed and co-staining of BLM was carried out with antibodies against pSer1981ATM and NBS1.

C. Time-dependent increase in the colocalization between BLM with γH2AX foci after 4-OHT mediated DSB induction. U2OS-AsiSI-ER cells were grown either without 4-OHT treatment (-) or treated with 4-OHT for the indicated time intervals. Staining was carried out with anti-BLM and anti-γH2AX antibodies. The number of BLM and γH2AX foci which colocalized per cell has been presented as mean ± S.D. p values from Student's t-test, $p \leq 0.05$.

D. BLM is recruited to the DSBs. U2OS AsiSI-ER cells were either transfected with siBLM or siControl. 44 hrs post-transfection cells were treated for 4 hrs with 4-OHT. ChIP was carried out using anti-BLM antibody. Recruitment of BLM to the indicated AsiSI generated DSBs or to the GAPDH loci were determined by ChIP-qPCR analysis.

E. BLM is recruited to the DSBs after sequence specific DSB induction. U2OS AsiSI-ER cells were either grown in untreated condition (-4-OHT) or treated with 4-OHT for 4 hrs. ChIP was carried out using either anti-BLM antibody or the corresponding IgG (mock). BLM recruitment at the indicated AsiSI generated DSB was determined by ChIP-qPCR analysis. Values presented are mean ± S.D. p values from Student's t-test. Four independent experiments carried out.

F, G. ML216 treatment increases the rate of SCE per metaphase. U2OS AsiSI-ER cells were grown in absence or presence of ML216. The number of SCEs per metaphase was calculated under each conditions. Representative metaphases for the two conditions are presented in (F) while quantitation of the number of SCEs per metaphase is presented in (G). Data in (G) was analyzed by unpaired two tailed Student's t-test with * denoting $p \leq 0.05$, obtained from three independent experiments.

Supplementary Figure 3
(Tripathi et al.)

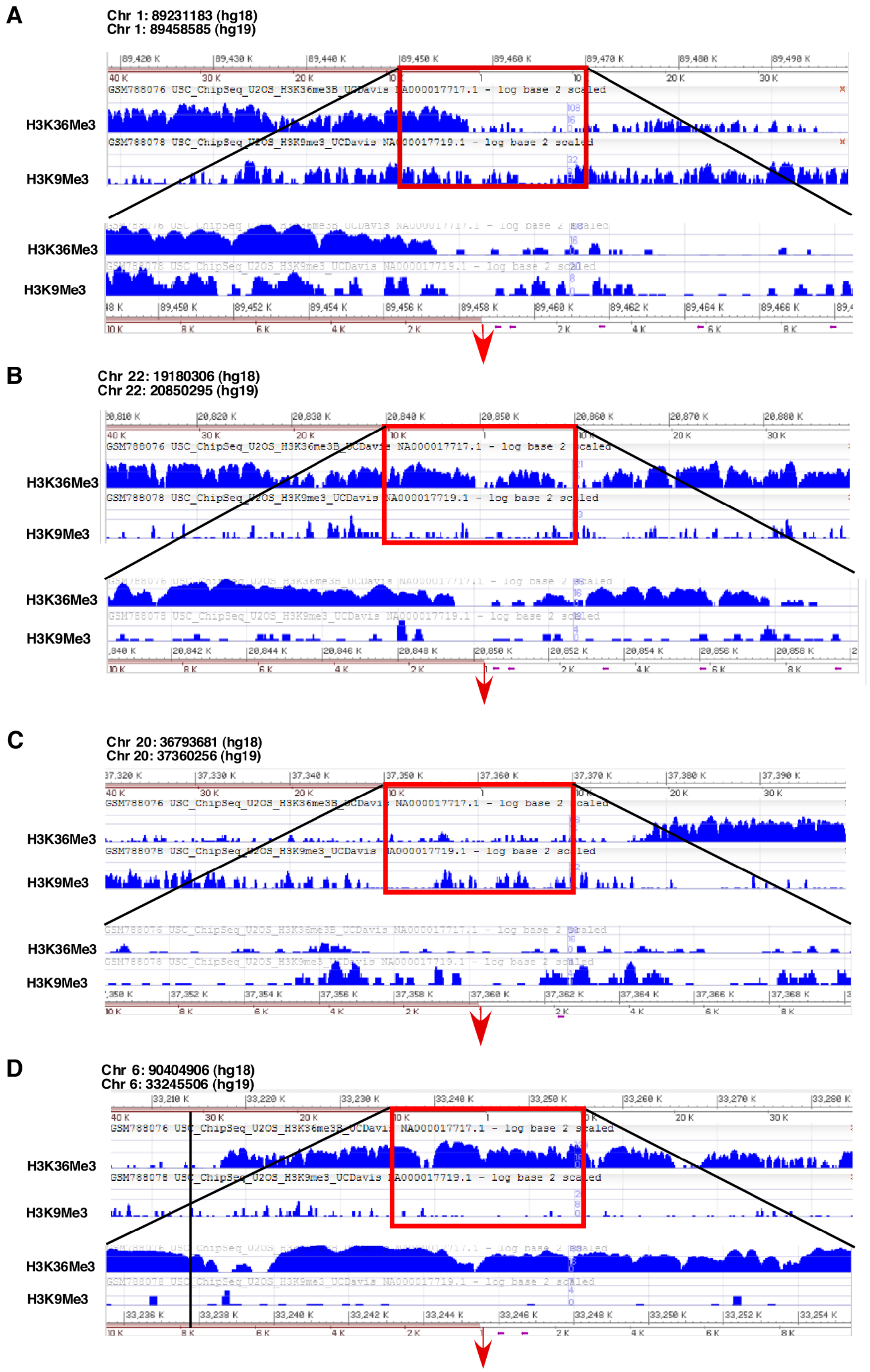

Epigenetic modifications around AsiSi sites. ChIP-seq data of H3K36me3 (associated with active chromatin) and H3K9me3 (associated with inactive chromatin) in U2OS cells were retreived from the ENCODE project. For (A) Chr 1: 89231183 (hg18) Chr 1: 89458585 (hg19); (B) Chr 22: 19180306 (hg18) Chr 22: 20850295 (hg19); (C) Chr 20: 36793681 (hg18) Chr 20: 37360256 (hg19); (D) Chr 6: 90404906 (hg18) Chr 6: 33245506 (hg19) the recruitment of the two histone marks 40 kb regions flanking each AsiSi site have been shown (top panels). The immediate 10 kb flanking the AsiSI sites have been further zoomed in (bottom panels). Red arrows indicate the AsiSI sites, while violet lines indicate the approximate positions of the primers used in ChIP-qPCR for the recruitment studies being reported.

Supplementary Figure 4
(Tripathi et al.)

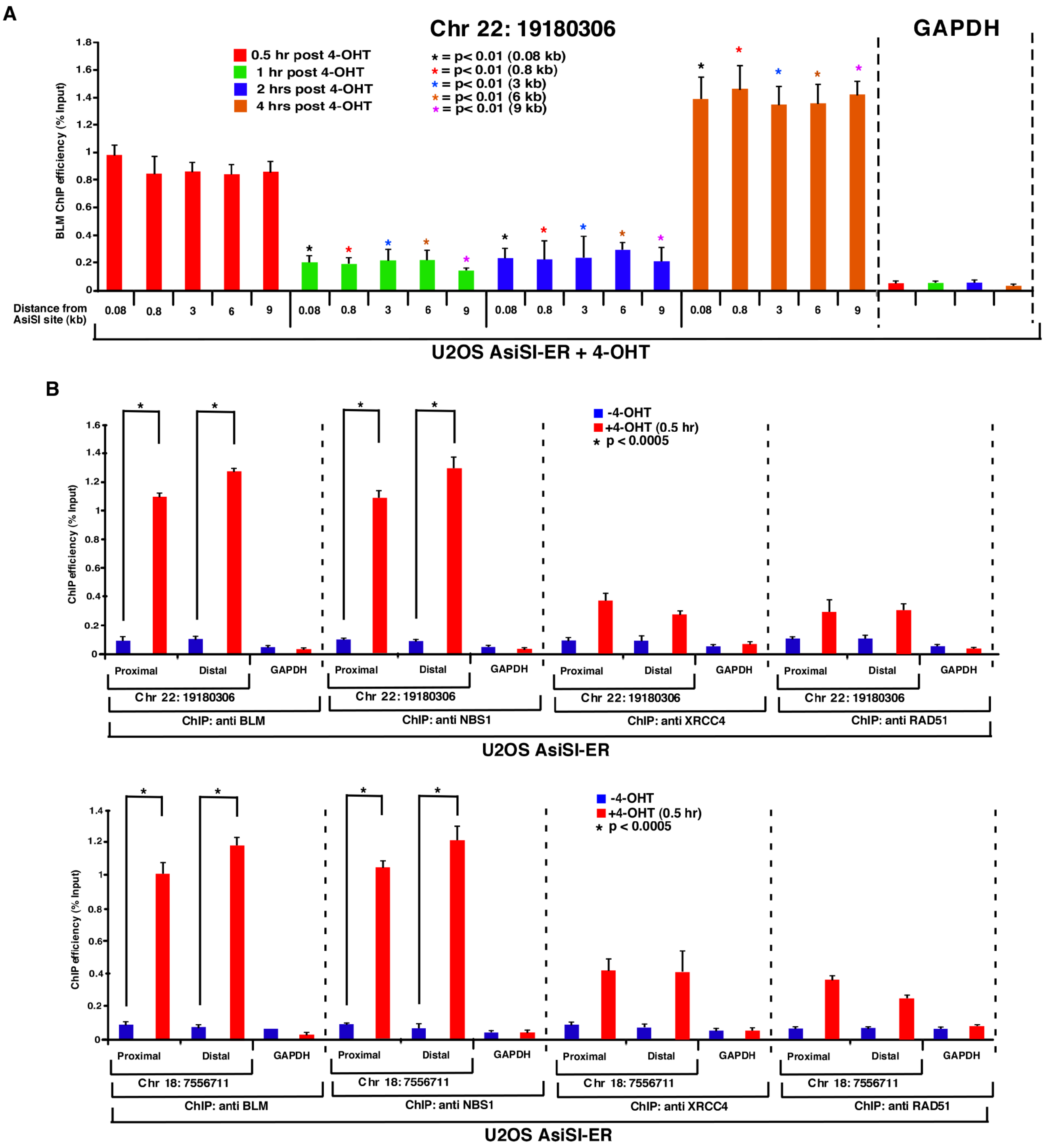

A. Biphasic BLM recruitment occurs at both proximal and distal positions with respect to the AsiSI site. U2OS AsiSI-ER cells were treated with 4-OHT for 0.5 hr, 1 hr, 2 hr, 4 hr. ChIP was carried out using anti-BLM antibody. BLM recruitment to the indicated AsiSI induced DSB was determined by ChIP-qPCR which was carried out using primers which were progressively distal from the AsiSI sites. Distance from AsiSi site at which BLM recruitment was measured was approximately 0.08 kb, 0.8 kb, 3 kb, 6 kb, 9 kb.

B. BLM is co-recruited to the DSBs with NBS1 but not with DNA repair proteins in early phase of recruitment. Same as (A) except U2OS AsiSI-ER cells were treated with 4-OHT for 0.5 hr. Parallel ChIPs with anti-BLM, anti-NBS1, anti-XRCC4 and anti-RAD51 antibodies. Recruitment of BLM, NBS1, XRCC4 and RAD51 to the indicated DSBs or to the GAPDH loci was determined by carrying out ChIP-qPCR analysis. Values presented are mean ± S.D. p values from Student’s t-test. Four independent experiments carried out.

Supplementary Figure 5
(Tripathi et al.)

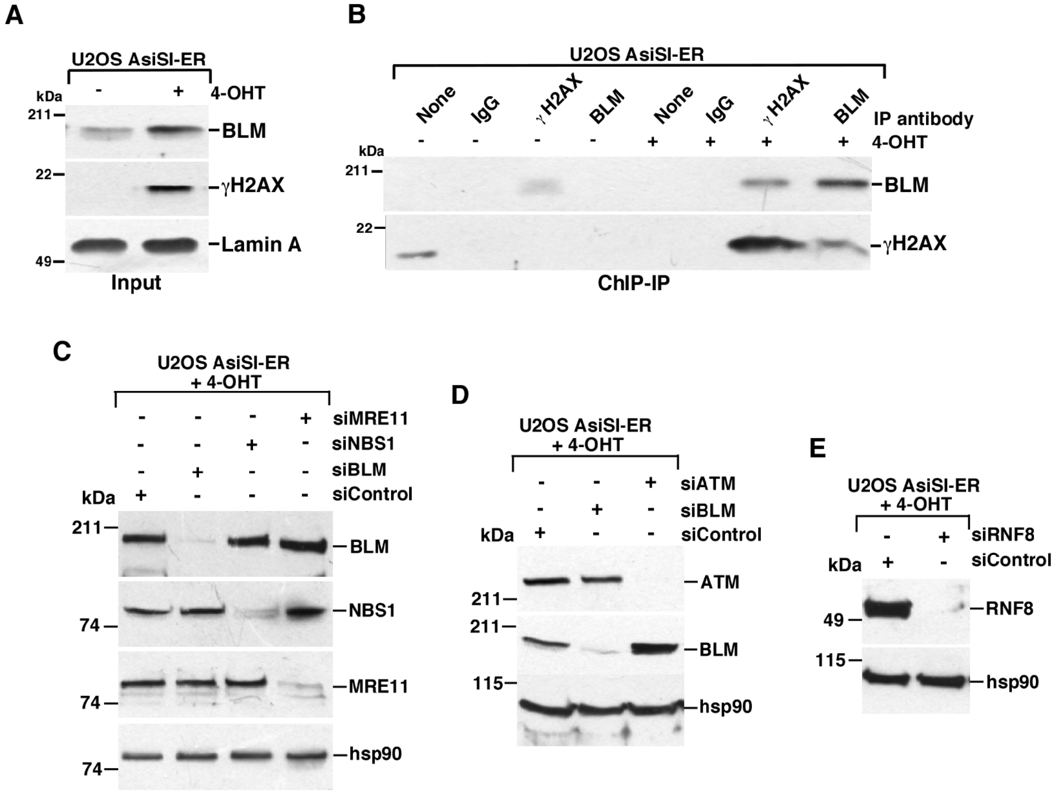

A, B. BLM and γH2AX interact at the DSBs. (A) U2OS AsiSI-ER cells were either grown in asynchronous condition (-4-OHT) or treated with 4-OHT (+4-OHT). Western blots were carried out with ChIP elutes with antibodies against BLM, γH2AX and Lamin A. (B) Immunoprecipitations were carried out with antibodies against BLM or γH2AX or the corresponding IgG with the ChIP eluates (in both ±4-OHT conditions). The immunoprecipitates were probed with antibodies against BLM and γH2AX.

C-E. BLM, NBS1, MRE11 and RNF8 are depleted by cognate siRNAs. U2OS AsiSI-ER cells were transfected with either siControl or siRNAs against (C) BLM, NBS1, MRE11, (D) BLM and ATM (E) RNF8. All transfections were carried out in presence of 4-OHT for 4 hrs. Lysates made were probed with antibodies against (C) BLM, NBS1, MRE11, hsp90 (D) ATM, BLM, hsp90, (E) RNF8, hsp90.

Supplementary Figure 6
(Tripathi et al.)

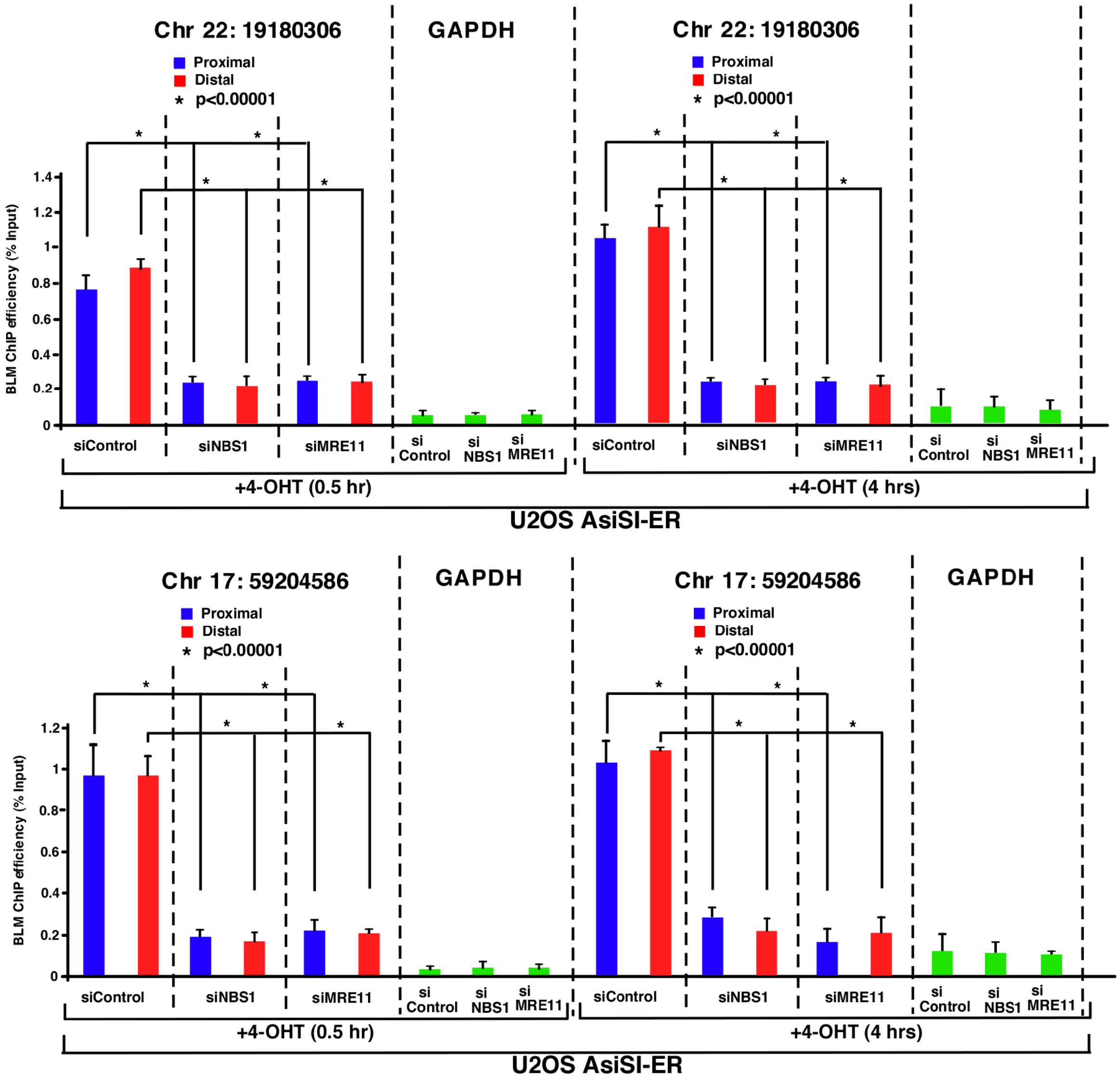

Recruitment of BLM to DSBs depends on NBS1 and MRE11 in both early and late phase. U2OS AsiSI-ER cells were either transfected with siControl or siNBS1 or siMRE11. 44 hrs post-transfection cells were treated for either 0.5 hr or 4 hrs with 4-OHT. ChIP was carried out using anti-BLM antibody. Recruitment of BLM to the indicated two AsiSI generated DSBs or the GAPDH loci were determined by ChIP-qPCR analysis. Values presented are mean ± S.D. p values from Student's t-test. Four independent experiments carried out.

Supplementary Figure 7
(Tripathi et al.)

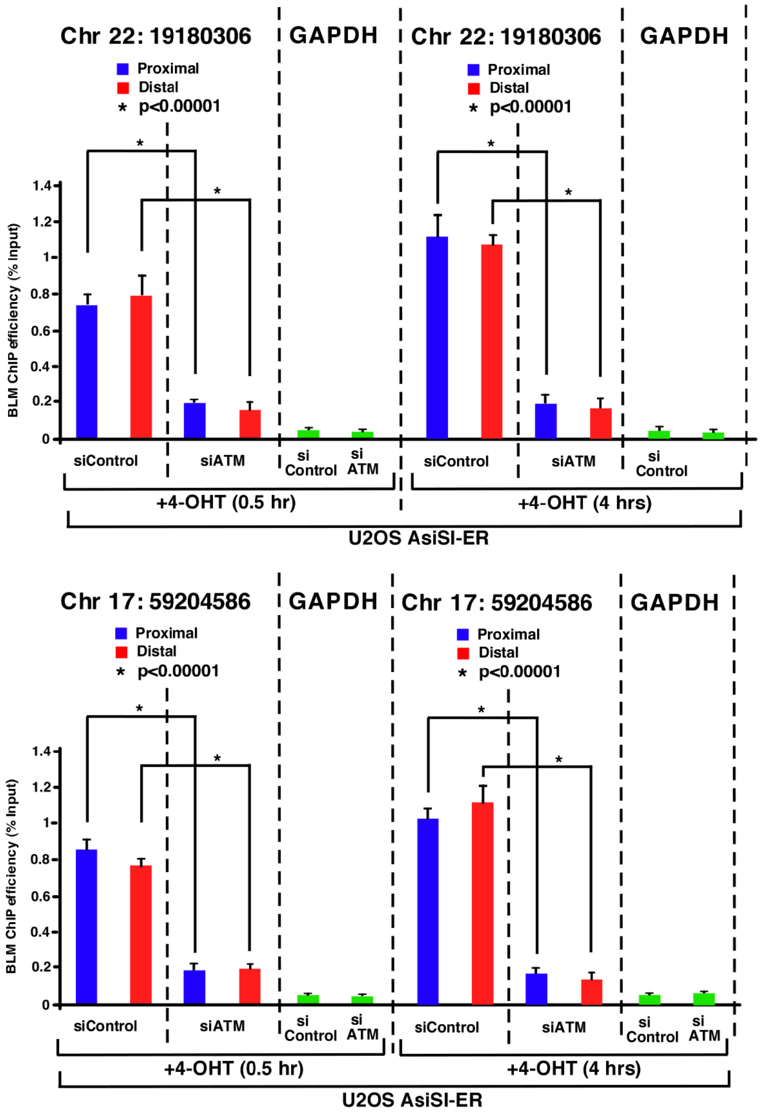

Recruitment of BLM to DSBs depends on ATM in both early and late phase. U2OS AsiSI-ER cells were either transfected with siControl or siATM. 44 hrs post-transfection cells were treated for either 0.5 hr or 4 hrs with 4-OHT. ChIP was carried out using anti-BLM antibody. Recruitment of BLM to the indicated two AsiSI generated DSBs or the GAPDH loci were determined by ChIP-qPCR analysis. Values presented are mean ± S.D. p values from Student's t-test. Four independent experiments carried out.

Supplementary Figure 8
(Tripathi et al.)

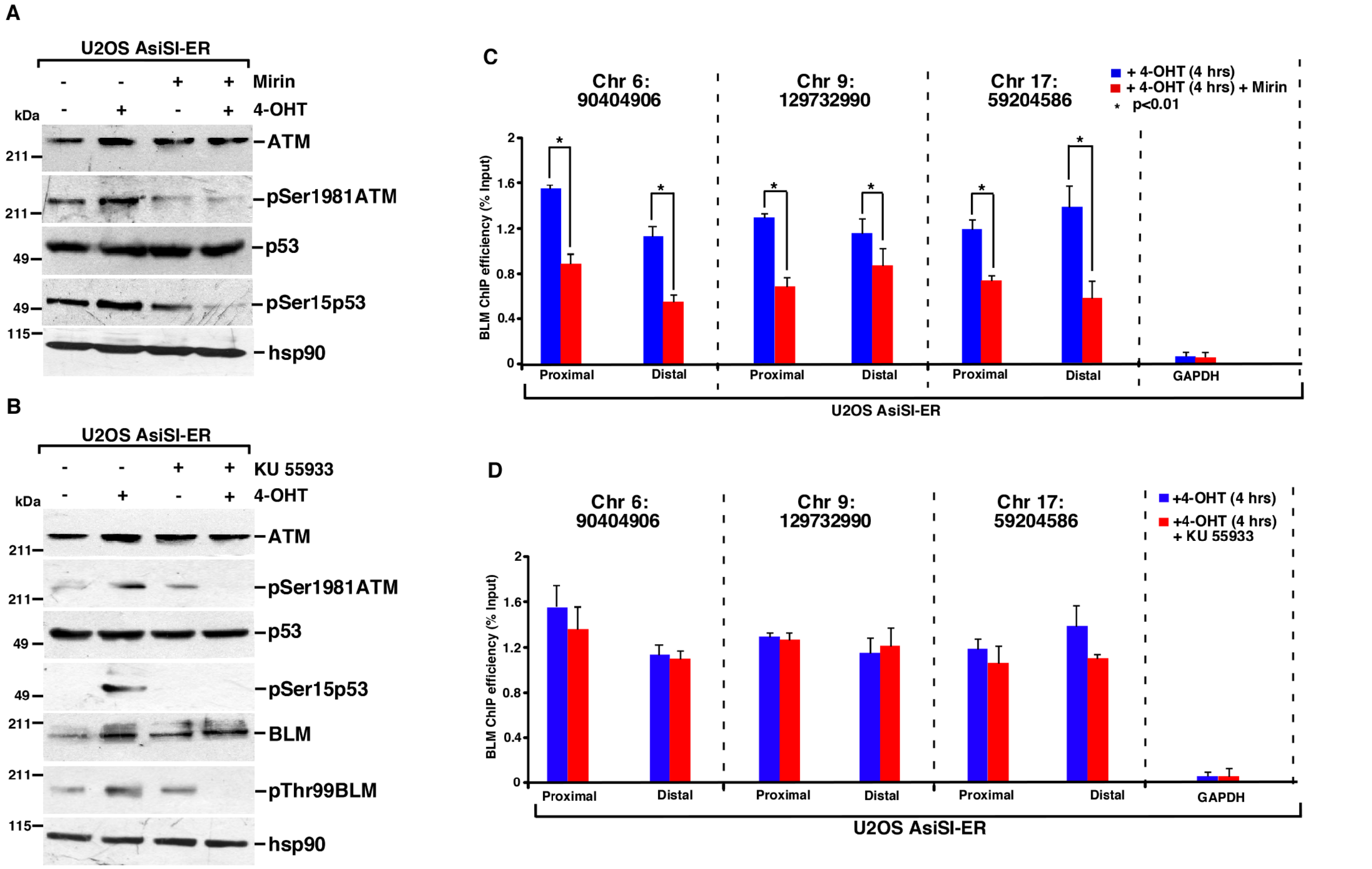

A, B. Validation of the potency of Mirin and KU 55933. U2OS AsiSI-ER cells, grown in absence or presence of 4-OHT (4 hrs), were co-treated with (A) Mirin or (B) KU 55933. The lysates made were probed with antibodies against (A) ATM, pSer1981ATM, p53, pSer15p53, hsp90 (B) ATM, pSer1981ATM, p53, pSer15p53, BLM, pThr99BLM, hsp90.

C. Recruitment of BLM to DSBs in late phase depends on MRE11 activity. U2OS AsiSI-ER cells were grown either in presence of only 4-OHT (+4-OHT, 4 hrs) or in concurrrent presence of both 4-OHT and Mirin. ChIP was carried out with anti-BLM antibody on chromatin obtained from cells grown in the above mentioned conditions. Recruitment of BLM to the indicated DSBs or to the GAPDH loci were determined by ChIP-qPCR.

D. Recruitment of BLM to DSBs in late phase does not depend on ATM activity. U2OS AsiSI-ER cells were grown either in presence of only 4-OHT (+4-OHT, 4 hrs) or in concurrrent presence of both 4-OHT and KU 55933. ChIP was carried out with anti-BLM antibody on chromatin obtained from cells grown in the above mentioned conditions. Recruitment of BLM to the indicated DSBs or to the GAPDH loci were determined by ChIP-qPCR. Values presented are mean ± S.D. p values from Student's t-test. Four independent experiments carried out.

Supplementary Figure 9
(Tripathi et al.)

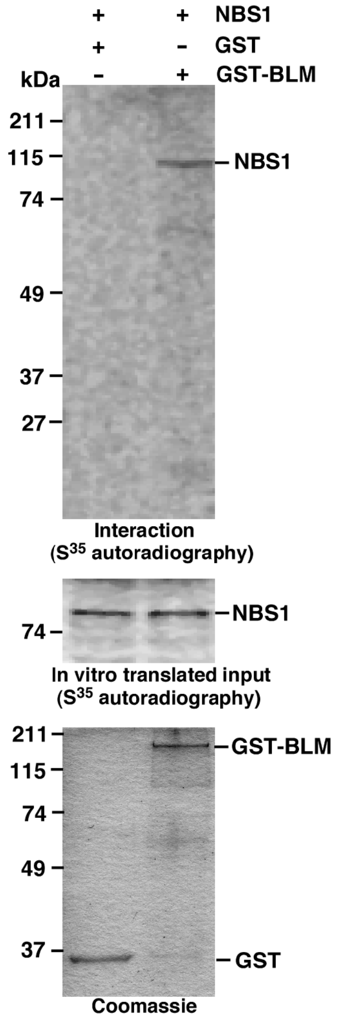

Recombinant BLM interacts with NBS1. In vitro interaction was carried out between Glutathione Sepharose bound recombinant GST-BLM or GST (bottom) with S^{35} methionine radiolabelled NBS1 (middle). Post-interaction the radioactivity to the bead bound proteins was detected by autoradiography after 7 days of exposure (top).

Supplementary Figure 10
(Tripathi et al.)

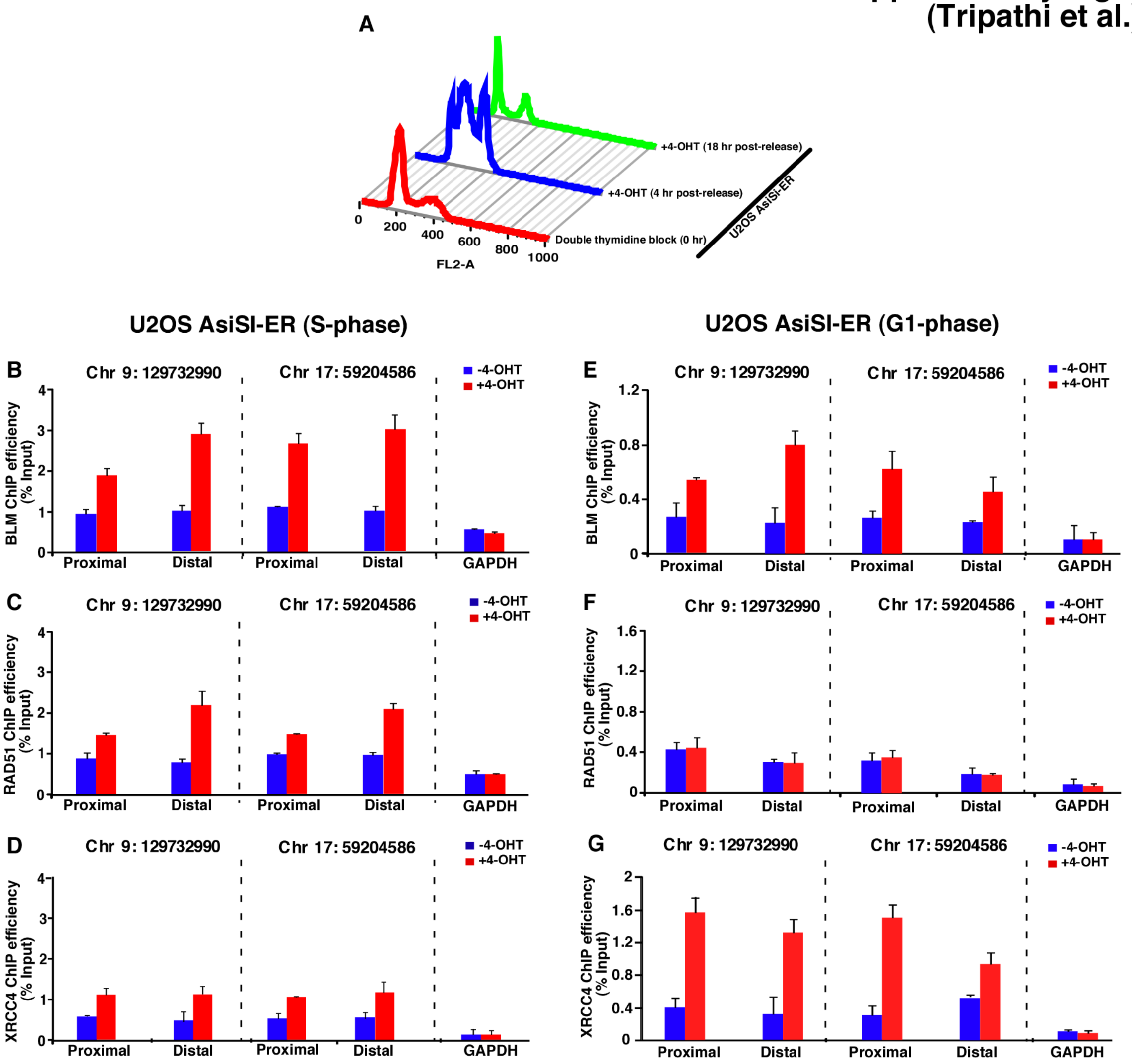

A. Synchronization of U2OS AsiSI-ER cells in S- and G1 phases of the cell cycle. U2OS AsiSI-ER cells, after double thymidine block, were released in normal growth medium for the indicated time intervals in presence of 4 hrs of 4-OHT treatment. The cell cycle profile at each time interval was obtained by subjecting the cells to flow cytometry analysis.

B-D. Recruitment of BLM, RAD51, XRCC4 to the DSBs in S-phase. U2OS AsiSI were synchronized in S-phase (4 hrs post-release from double thymidine block). Chromatin was prepared from these S-phase cells grown without (-4-OHT) or after 4 hrs of 4-OHT treatment (+4-OHT). ChIP was carried out with antibodies against (B) BLM, (C) RAD51, (D) XRCC4. Recruitment to the indicated AsiSI induced DSBs or to the GAPDH loci were determined by ChIP-qPCR.

E-G. BLM and XRCC4 are co-recruited to the DSBs in G1-phase. U2OS AsiSi-ER cells were synchronized in G1-phase (18 hrs post-release from double thymidine block). Chromatin was prepared from these G1-phase cells grown without (-4-OHT) or after 4 hrs of 4-OHT treatment (-4-OHT). ChIP was carried out with antibodies against (E) BLM, (F) RAD51, (G) XRCC4. Recruitment to the indicated AsiSI induced DSBs or to the GAPDH loci were determined by ChIP-qPCR. Values presented are mean ± S.D. p values from Student's t-test. Four independent experiments carried out.

Supplementary Figure 11
(Tripathi et al.)

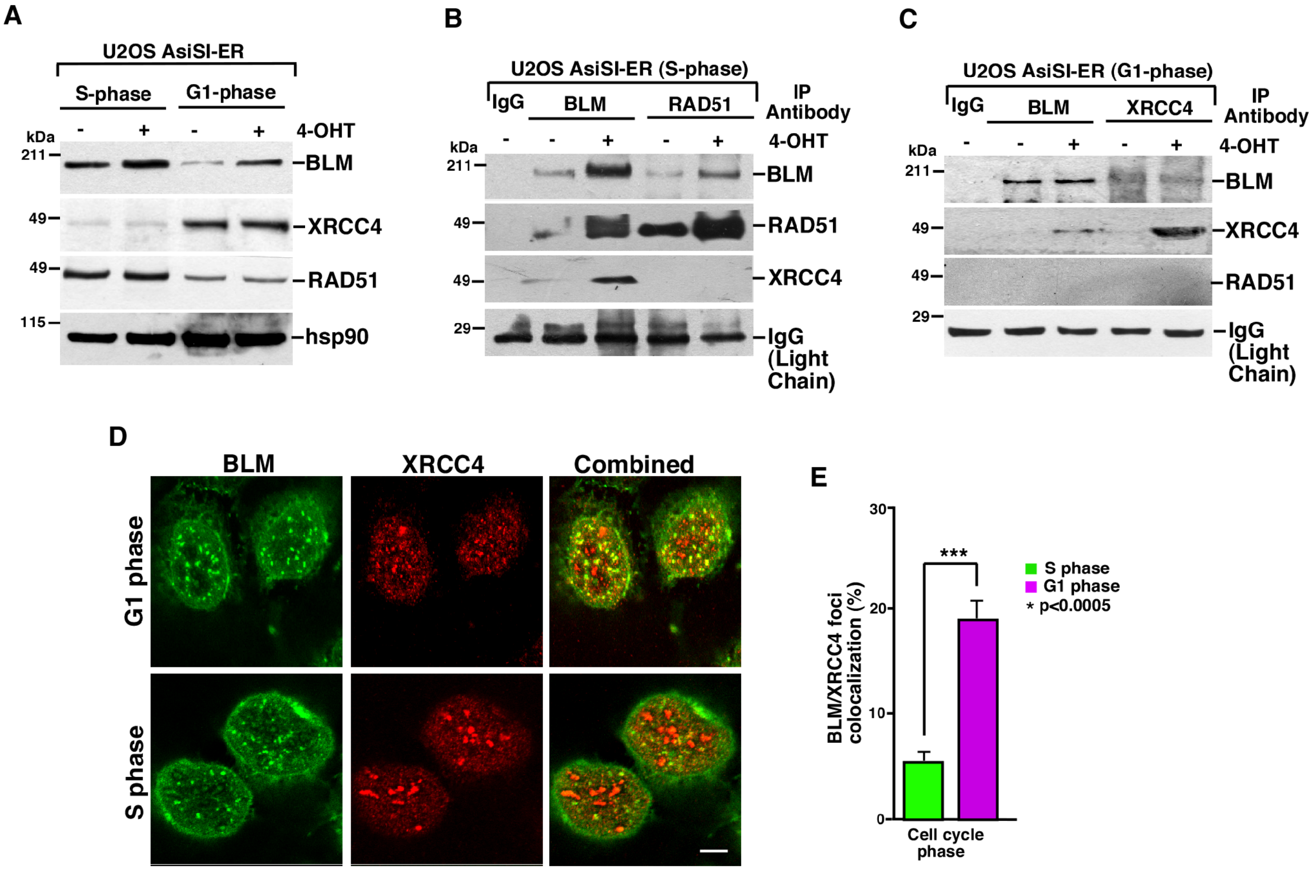

A-C. BLM interacts with RAD51 and XRCC4 in S- and G1-phase. (A) Levels of BLM, XRCC4 and RAD51 were determined in U2OS AsiSI-ER cells synchronized in either S- or G1 phase. Cells were grown either in absence or presence of 4-OHT treatment for 4 hrs. The lysates made were subjected to western analysis with antibodies against BLM, XRCC4, RAD51 and hsp90. (B, C) Immunoprecipitations were carried out with (B) anti-BLM and anti-RAD51 antibodies with lysates from S-phase or with (D) anti-BLM and anti-XRCC4 antibodies with lysates from G1 phase. Westerns were carried out with antibodies against BLM, RAD51 and XRCC4. IgG indicates the equal amounts of antibodies used for immunoprecipitations.

D, E. BLM colocalizes with XRCC4 in G1- and S-phase to different extent. U2OS AsiSI cells synchronized in S- or G1-phase were immunostained with antibodies against BLM and XRCC4. Imaging was done by confocal microscopy. Representative cells are shown in (D). Bar, 5µm. The percentage of BLM foci colocalization with XRCC4 in S and G1 phase is shown in (E). Values mean ± S.D.

Supplementary Figure 12
(Tripathi et al.)

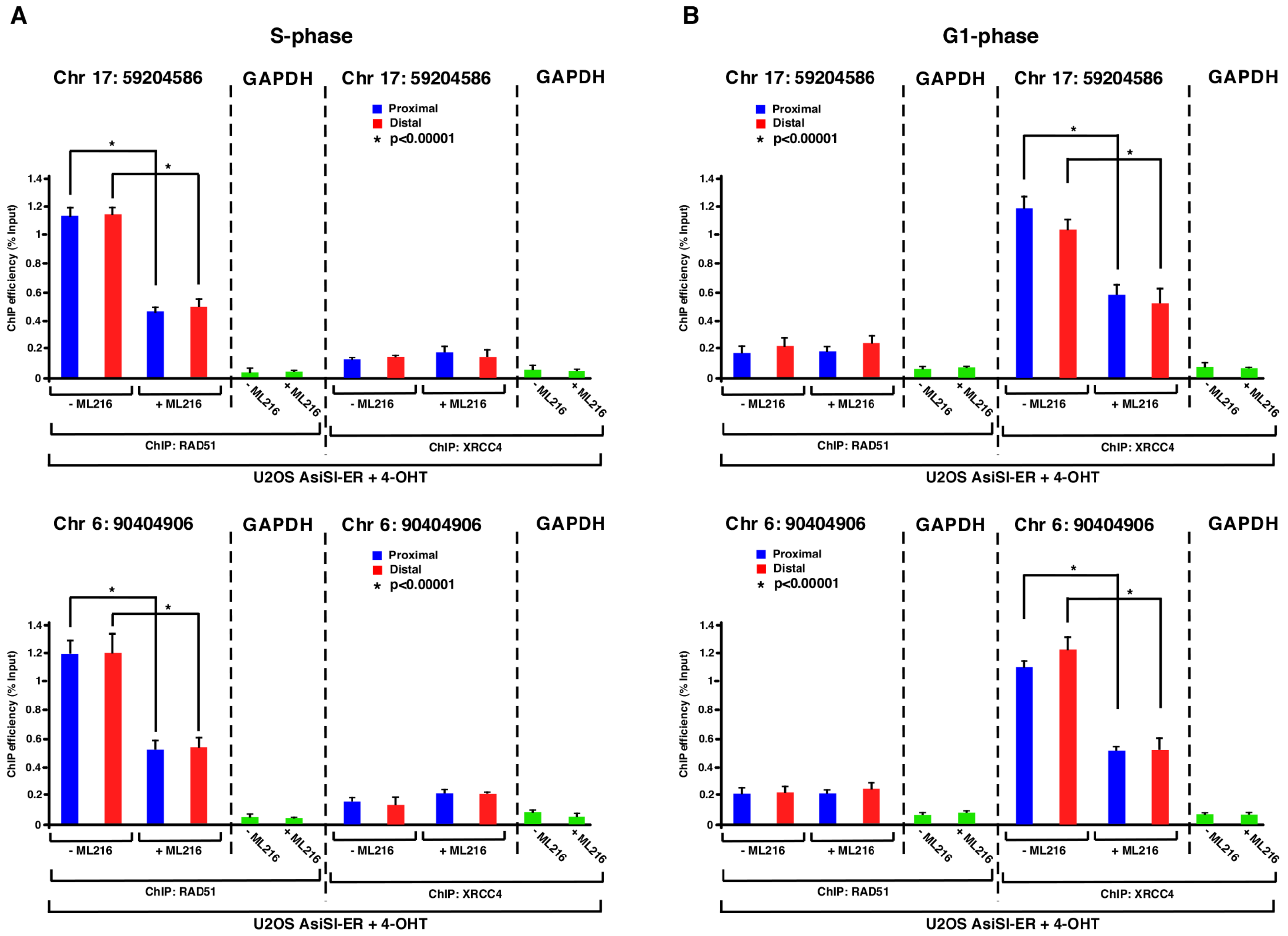

Recruitment of RAD51 and XRCC4 to the DSBs in S- and G1-phase depends on the helicase activity of BLM. U2OS AsiSI-ER cells were synchronized either in (A) S-phase or (B) G1 phase. Cells in either of the phases were subjected to 4 hrs of 4-OHT treatment, carried out either in absence or presence of ML216. Parallel ChIPs were carried out with antibodies against RAD51 and XRCC4. The recruitment of RAD51 and XRCC4 to the indicated AsiSI induced DSBs or to the GAPDH loci was determined by ChIP-qPCR. Values presented are mean ± S.D. p values from Student's t-test. Four independent experiments carried out.

Supplementary Figure 13
(Tripathi et al.)

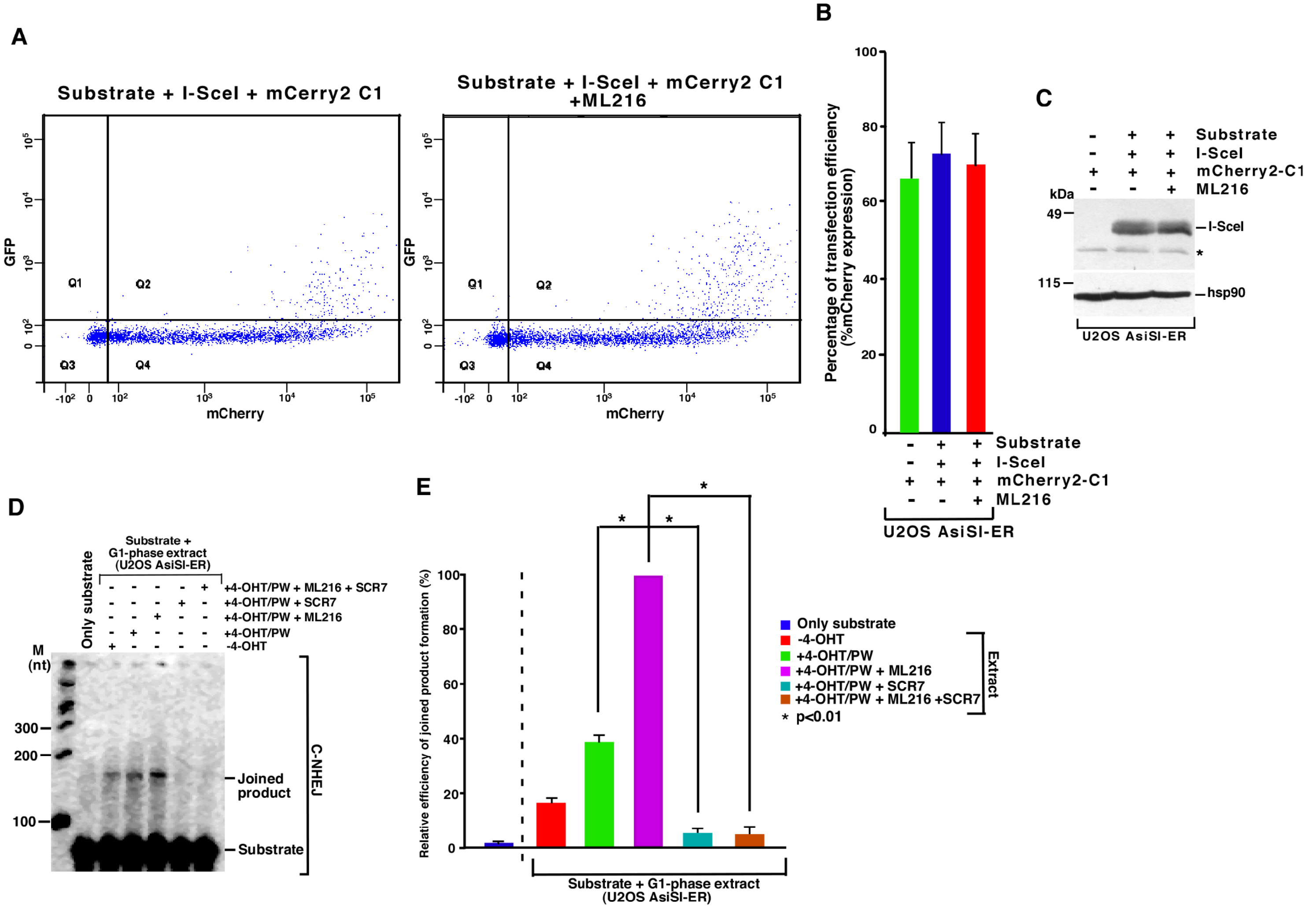

A, B. Total NHEJ is increased in absence of BLM helicase activity. Interchromosomal substrate (pJS296) and I-SceI expression construct were co-transfected with mCherry2 C1 (to measure transfection efficiency) in asynchronous U2OS AsiSI-ER cells (grown in absence of 4-OHT). Cells were allowed to grow either asynchronously or treated with ML216. Cells were harvested after 36 hrs and sorted for GFP (measure of c-NHEJ) and mCherry. Typical gated scatter plots are shown in (A). The relative percentage of mCherry transfection in different conditions, as determined by FACS analysis, is represented in (B). Values presented are mean ± S.D. Six independent experiments carried out. (C) Western blots were carried out with lysates made in the above conditions with antibodies against I-SceI and hsp90. A cross-reactive band is represented by *.

D, E. Increase in c-NHEJ activity due to ML216 treatment is mediated via Ligase IV. Extracts were prepared from S-phase arrested U2OS AsiSi ER cells grown in -4-OHT, +4-OHT/PW, +4-OHT/PW +ML216, +4-OHT/PW + SCR7, +4-OHT/PW +ML216 +SCR7 conditions. c-NHEJ assays were carried out with the respective extracts. (D) Representative autoradiogram of the c-NHEJ assay. (E) Percentage efficiency of joint product formation is represented.The product formed in +4-OHT/PW +ML216 is taken as 100% and all other products are calculated relative to the above condition. Values presented are mean ± S.D. p values from Student's t-test. Three independent experiments carried out.

Supplementary Figure 14
(Tripathi et al.)

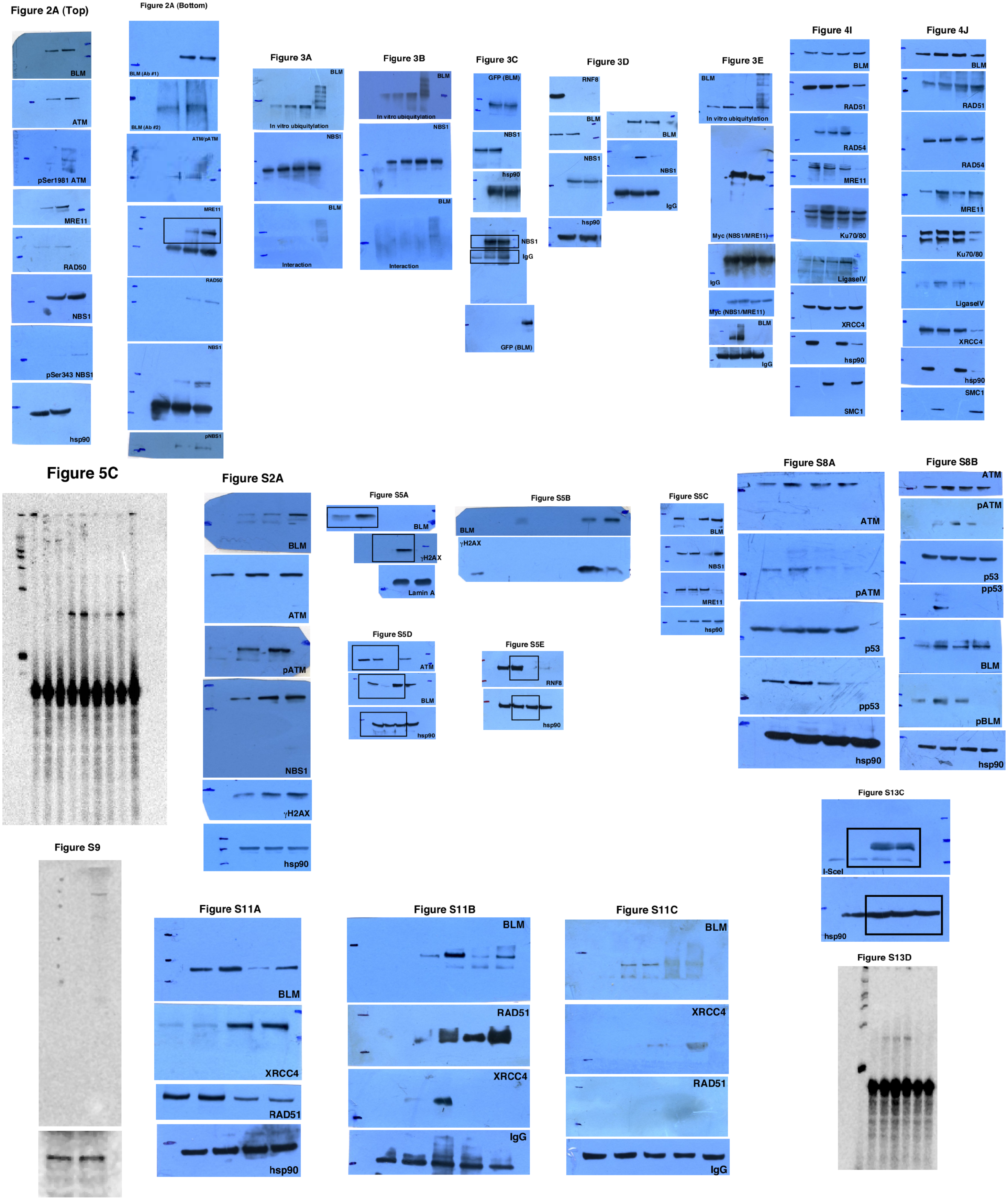

Figure-wise source file of all the western blots and autoradiograms presented in this work.

Supplementary Table 1: List of ChIP-qPCR primers used to study BLM recruitment at AsiSI sites

Experimental Primers (5' – 3')					
**Chromosome number**	**AsiSI site position (hg18)**	**Proximal**		**Distal**	
		**Forward Primer (distance from AsiSI site)**	**Reverse Primer (distance from AsiSI site)**	**Forward Primer (distance from AsiSI site)**	**Reverse Primer (distance from AsiSI site)**
Chr. 1	89231183	CCT GGA TAT GAG TTT GAT CAG C (486 nt)	CTC TCC TTT CGC TGA CAC TG (625 nt)	AGG AAT TGA CTG CGG TGT TC (1255 nt)	GGG GAG GAG GAA AGG TGT AG (1027 nt)
Chr. 22	19180306	GCC ACC TTC GAT ATT TTC TCC AG (5233 nt)	GTT GTT TTT GCA GGA TTT TGA GTC C (5465 nt)	GCC GAG ATG TCA CCA CAG CAC (10126 nt)	GAT TAC AAG CGT ACC CCA CCA TG (10344 nt)
Chr. 9	36248512	CAC ATG GCA CCT CTG ACT TCT G (2547 nt)	TGC AGA TGT GAC CAG GAC TTT GAG A (2672 nt)		
Chr. 18	7558351	GGT TCT CTT TAG GCC AAT GGA CTA (2278 nt)	GAG GAC AGG AGA GCA CTG CAG AA (2385 nt)		
Chr. 20	36793681	GTT TAC CTG AAG CTG TGG AAC ATG (1950 nt)	TTG AGC ACT AGG GGC TGT TAG CT (2058 nt)		
Chr. 21	32167388	CAG GCC ATT TCA GAG ACT AGG ACT (2319 nt)	GGG TTT GAT TCA AGT TCA CTG CTA A (2215 nt)		
Chr. 18	7556711	TCC CCT GTT TCT CAG CAC TT (243 nt)	CTT CTG CTG TTC TGC GTC CT (87 nt)	GGA GAA GTG GCA GGA CAA TG (879 nt)	CAA GGC AAA TTT GGG GAC TA (714 nt)
Chr. 6	90404906	ATC GGG CCA ATC TCA GAG G (57 nt)	GCG ACG CTA ACG TTA AAG CA (152 nt)	TTT TTG GGG GAA AGA GGT G (710 nt)	AGT GGG TGA GCC ATT CAA AG (931 nt)
Chr. 9	129732990	GTC CCT CGA AGG GAG CAC (88 nt)	CCG ACT TTG CTG TGT GAC C (288 nt)	TAT GGG ACC AAG CGA GTA GG (864 nt)	GCC TCA CAC ACA CAC CCA TA (1108 nt)
Chr. 17	59204586	CCG TCC GTT ACG TAG AAT GC (92 nt)	GGG CGG GGA TTA TGT AAT TT (222 nt)	GGG ACA GCG CGT ACT TTG (803 nt)	TCG CTA GGC CCA GCA GTT (916 nt)

Control Primers (5' – 3')		
**Name of control gene**	**Forward Primer**	**Reverse Primer**
GAPDH	GCA GCC CCT TCA TAC CCT CAC GT	GAG CCA CAC CAT CCT AGT TGC

Supplementary Table 2: List of ChIP-qPCR primers used to study BLM recruitment at different distances from the AsiSI site

Experimental Primers (5' – 3')				
**Chromosome number**	**AsiSI site position(hg18)**	**Designation**	**Forward Primer (distance from AsiSI site)**	**Reverse Primer (distance from AsiSI site)**
Chr. 1	89231183	80bp	GGA AGG GGA CAA GAT GGA GGA (57 nt)	ATG TGA TCC CGG CCG ACT C (139 nt)
		800bp	CCT GGA TAT GAG TTT GAT CAG C (486 nt)	CTC TCC TTT CGC TGA CAC TG (625 nt)
		3kb	CAC TCC AGC TCT GGA CCA AT (2914 nt)	ACT GTG GCT ACA CTT CAG GAA A (2992 nt)
		6 kb	AGG AAA AAT AAA AAT TCT GGC CAC T (5935 nt)	GCA ACA TGC AGT CAG CAT CC (6019 nt)
		9 kb	GGT AGG GGC CAG GGA ATC TT (9008 nt)	AAA GCT CAC TGT CCA AGG AGG (9090 nt)
Chr. 22	19180306	80bp	ACC AAC GAA TGA GCG AAT TT (54 nt)	GGG AAT GAG GAA AGG AGA GG (190 nt)
		800bp	GGT GCC GCT GAC CGT A (589 nt)	TCA GTA AAG GAA CGG GCA CA (671 nt)
		3kb	TTC TTG TCC CTT CCT TTC CA (2838 nt)	AAA GCC AAG CTC TCA GGA CA (2988 nt)
		6 kb	GCC ACC TTC GAT ATT TTC TCC AG (5233 nt)	GTT GTT TTT GCA GGA TTT TGA GTC C (5465 nt)
		9 kb	GCC GAG ATG TCA CCA CAG CAC (10126 nt)	GAT TAC AAG CGT ACC CCA CCA TG (10344 nt)

Control Primers (5' – 3')		
**Name of control gene**	**Forward Primer**	**Reverse Primer**
GAPDH	GCA GCC CCT TCA TAC CCT CAC GT	GAG CCA CAC CAT CCT AGT TGC

Supplementary Table 3: Accuracy of end joining close to AsiSI induced DSBs upon +4-OHT treatment alone or in combination with ML216

	Chr 18: 7556711		Chr 6: 90404906		Chr 9: 129732990		Chr 17: 59204586	
	+4-OHT	+4-OHT +ML216	+4-OHT	+4-OHT +ML216	+4-OHT	+4-OHT +ML216	+4-OHT	+4-OHT +ML216
Number of clones sequenced	35	35	35	35	35	35	35	35
Deletions ≥ 1	5	12	3	7	4	8	5	11
	14.29%	34.29%	8.57%	20%	11.43%	22.86%	14.29%	31.43%
Insertions ≥ 1	4	7	3	5	3	6	3	6
	11.43%	20%	8.57%	14.29%	8.57%	17.14%	8.57%	17.14%
Deletion with insertions	2	5	0	2	2	4	0	3
	5.71%	14.29%	0%	5.71%	5.71%	11.43%	0%	8.57%

On both sides of any AsiSI site, a minimum of 50 nucleotides and a maximum of 100 nucleotides were considered to determine the accuracy of the end joining. Deletions, insertions and deletions with insertions are calculated from positive clones obtained over three independent experiments.

Supplementary Table 4: List of antibodies used in the study

**Name of antibody**	**Reference**	**Application(s) and dilutions**
Anti-BLM	A300-110A (Bethyl Laboratories)	ChIP (2μg/ChIP), WB (except ubiquitylation blots, 1:2500), IP (1μg/IP)
	A300-120A (Bethyl Laboratories)	WB (only ubiquitylation blots) (1:1000)
	sc-7790 (Santa Cruz Biotechnology)	IF (1:200)
Anti-pThr99BLM	ab62206 (Abcam)	WB (1:1000)
Anti-γH2AX	ab26350 (Abcam)	IF (1:300), IP (1μg/IP), WB (1:1000)
Anti-RAD51	sc-8349 (Santa Cruz Biotechnology)	ChIP (2μg/ChIP), WB (1:2000)
Anti-RAD54	ab10705 (Abcam)	WB (1:5000)
Anti-NBS1	NB100-143 (Novus Biologicals)	ChIP (2μg/ChIP), WB (1:2000)
Anti-pSer343 NBS1	#3001 (Cell Signaling Technology)	WB (1:500)
Anti-MRE11	NB100-142 (Novus Biologicals)	WB (1:1000)
Anti-RAD50	611010 (BD Transduction Laboratories)	WB (1:1000)
Anti-Ku70	sc-9033 (Santa Cruz Biotechnology)	WB (1:1000)
Anti-Ku86	sc-9034 (Santa Cruz Biotechnology)	WB (1:1000)
Anti-Ligase IV	sc-11748 (Santa Cruz Biotechnology)	WB (1:1000)
Anti-XRCC4	ab145 (Abcam)	ChIP (2μg/ChIP), WB (1:1000), IF (1:200)
Anti-myc tag	#2278 (Cell Signaling Technology)	IP (1μg/IP), WB (1:1000)
Anti-ATM	sc-1213 (Santa Cruz Biotechnology)	WB (1:750)
Anti-pSer1981ATM	sc-47739 (Santa Cruz Biotechnology)	WB (1:1000)
Anti-p53	sc-126 (Santa Cruz Biotechnology)	WB (1:2000)
Anti-pSer15p53	#9284 (Cell Signaling Technology)	WB (1:500)
Anti-hsp90α/β	sc-7947 (Santa Cruz Biotechnology)	WB (1:2000)
Anti-RNF8	sc-271462 (Santa Cruz Biotechnology)	WB (1:1000)
Anti-Lamin A/C	#05-714 (Millipore)	WB (1:5000)
Anti-GFP	sc-9996 (Santa Cruz Biotechnology)	WB (1:3000), IP (1μg/IP)
Anti-SMC1	ab9262 (Abcam)	WB (1:2000)
Anti-I-SceI	ab216263 (Abcam)	WB (1:1000)

WB: Western blotting; IP: Immunoprecipitation; IF: Immunofluorescence
ChIP: Chromatin immunoprecipitation

Supplementary Table 5: List of siRNAs used in the study

Name of cognate gene	Sequence directed against	Past reference for usage and validation
siBLM	AGC AGC GAU GUG AUU UGC A	1
siRNF8	GGA CAA UUA UGG ACA ACA A	1
siATM	AAC AUA CUA CUC AAA GAC AUU	2
siMRE11	GCU AAU GAC UCU GAU GAU A	3
siNBS1	GCA GUU CAG UCC AAG AAG C	4
siControl	D-001810-01-05 (Dharmacon)	1

1 Tikoo, S. *et al.* Ubiquitin-dependent recruitment of the Bloom Syndrome helicase upon replication stress is required to suppress homologous recombination. *EMBO J* **32**, 1778-1792, doi:emboj2013117 [pii] 10.1038/emboj.2013.117 (2013).

2 Andreassen, P. R., D'Andrea, A. D. & Taniguchi, T. ATR couples FANCD2 monoubiquitination to the DNA-damage response. *Genes Dev* **18**, 1958-1963 (2004).

3 Myers, J. S. & Cortez, D. Rapid activation of ATR by ionizing radiation requires ATM and Mre11. *J Biol Chem* **281**, 9346-9350, doi:10.1074/jbc.M513265200 (2006).

4 Mochan, T. A., Venere, M., DiTullio, R. A., Jr. & Halazonetis, T. D. 53BP1 and NFBD1/MDC1-Nbs1 function in parallel interacting pathways activating ataxia-telangiectasia mutated (ATM) in response to DNA damage. *Cancer Res* **63**, 8586-8591 (2003).
